# Can visco-elastic phase separation, macromolecular crowding and colloidal physics explain nuclear organisation?

**DOI:** 10.1186/1742-4682-4-15

**Published:** 2007-04-12

**Authors:** Francisco J Iborra

**Affiliations:** 1 MRC Molecular Haematology Unit, Weatherall Institute of Molecular Medicine, John Radcliffe Hospital, Headington, Oxford, OX3 9DS, UK

## Abstract

**Background:**

The cell nucleus is highly compartmentalized with well-defined domains, it is not well understood how this nuclear order is maintained. Many scientists are fascinated by the different set of structures observed in the nucleus to attribute functions to them. In order to distinguish functional compartments from non-functional aggregates, I believe is important to investigate the biophysical nature of nuclear organisation.

**Results:**

The various nuclear compartments can be divided broadly as chromatin or protein and/or RNA based, and they have very different dynamic properties. The chromatin compartment displays a slow, constrained diffusional motion. On the other hand, the protein/RNA compartment is very dynamic. Physical systems with dynamical asymmetry go to viscoelastic phase separation. This phase separation phenomenon leads to the formation of a long-lived interaction network of slow components (chromatin) scattered within domains rich in fast components (protein/RNA). Moreover, the nucleus is packed with macromolecules in the order of 300 mg/ml. This high concentration of macromolecules produces volume exclusion effects that enhance attractive interactions between macromolecules, known as macromolecular crowding, which favours the formation of compartments. In this paper I hypothesise that nuclear compartmentalization can be explained by viscoelastic phase separation of the dynamically different nuclear components, in combination with macromolecular crowding and the properties of colloidal particles.

**Conclusion:**

I demonstrate that nuclear structure can satisfy the predictions of this hypothesis. I discuss the functional implications of this phenomenon.

## 

The cell exist a crowded environment of organelles, macromolecules, chromatin, membranes, and cytoskeletal filaments. The cell is not, however, simply a soup of its constituent parts, rather there exists an ordered structure referred to as compartmentalisation. Maintenance of compartmentalisation within the cell has fundamental implications for cellular function. In the cytoplasm, compartmentalisation is commonly achieved by confining macromolecules in lipid membranes thereby creating organelles such as mitochondria, lysosomes, Golgi apparatus, etc. However, even the cytoplasm regions not divided by membranes can show local differences in composition. Within the nucleus there also exist numerous distinct structures such as the nucleolus, interchromatin granule clusters (IGC), heterochromatin, and various bodies such as: Cajal, PML, SMN. Nuclear compartmentalization exists without any membranous division. Key questions such as how nuclear compartmentalization is achieved and why it exists, still remain unanswered. In a seminal paper Tom Misteli proposed self-organization as an explanation for the existence of nuclear compartmentalization [1] but the molecular basis for self-organization of nuclear structures is not fully understood. Another phenomenon implicated in nuclear compartment formation is macromolecular crowding, however, this only explains the existence of some of the nuclear structures [2], but is not enough to explain the different structures found in the cell nucleus. Several models have been proposed to explain three-dimensional chromatin organization, from modelling chromatin as balls connected by springs [3-5] to chromatin loops as semi flexible (self-avoiding) tubes [6]. All these models are very simplistic, tending to focus on chromatin as an independent entity floating in an ideal buffer. No consideration is given to the physical properties of the nuclear components and its consequences for nuclear structure. The main stumble block to date is no one model can fully account for the diversity of nuclear structures observed. Recent advances in biophysics have provided us with invaluable information and have allowed us to understand cell organization. In this paper I explore a biophysical explanation for compartmentalization within the cell nucleus.

## Dynamic Asymmetry within the Nucleus

Nuclear DNA is associated with histones, which are then packaged into an ordered structure called chromatin. This chromatin is further packaged into individual chromosomes that occupy distinct territories in the nucleus [7]. Within the mammalian nucleus, chromosomes territories show non-random, evolutionarily conserved radial organisation on the basis of gene content. Gene-rich chromosomes occupy a more internal nuclear location and gene-poor chromosomes reside at the nuclear periphery [8-10], which may be driven by the interaction of heterochromatin with the nuclear lamina [11]. While chromosome territories are more or less fixed throughout the cell cycle except for early in G1 [12] their constituent chromatin does show a degree of constrained diffusional motion. Chromatin dynamics in living cells have been studied by several groups by exploiting the lac operator/repressor system [13]. Integration of a *lac *operator array into the DNA of cells expressing GFP-*lac *repressor fusion protein allows chromatin movement to be monitored. The main findings of these studies are that chromatin moves in a Brownian manner with a diffusion coefficient in the range ~10^-4 ^to 10^-3 ^μm^2^/s [12,14]. Chromatin mobility is also affected by condensation state; euchromatin moves faster than heterochromatin [15].

Nuclear protein dynamics have also been studied extensively using photobleaching experiments, namely fluorescence recovery after photobleaching (FRAP), and fluorescence loss in photobleaching (FLIP). Experimental evidence shows that proteins are highly dynamic and move unrestricted through the nuclear volume in an energy-independent manner [1]. Whilst roving through the nuclear space a protein may engage with non-specific or high-affinity binding sites, as demonstrated by Phair et al [16] who estimate residence times of 2–30 s for chromatin proteins on both euchromatin and heterochromatin, and the time between binding events at around 100 ms. Some times diffusion is so rapid that FRAP or FLIP approaches are not suitable to calculate the diffusion coefficient. In these instances, diffusion measurements are performed using fluorescence correlation spectroscopy (FCS) [17]. This technique allows one to calculate the average time needed for a fluorescently labeled molecule to pass through a very small defined confocal volume. Since the confocal volume is a known measure the diffusion coefficient (D) of the molecule can be determined. Freely diffusible proteins within the nucleus move slower than in water but the D values are in the 0.2 to 20 μm^2^/s range [1,18]. In addition to proteins exhibiting highly dynamic diffusion, nuclear bodies also show a degree of motion. Gorisch et al. have studied the diffusion properties of both Cajal and PML bodies as well as a biochemically inactive body composed of murine Mx1 [19]. Their findings indicate that nuclear bodies show constrained diffusion within a chromatin corral, which can itself translocate.

The movement of RNA also can be measured by Fluorescent RNA Cytochemistry [20,21]. Photoactivation of caged fluorochromes conjugated to oligonucleotides allow of RNA molecules visualisation in living cells. In living cells the RNA demonstrates Brownian motion, with diffusion constants ranging from 0.1 to 10 μm^2^/s [20,22].

These observations show a nucleus with two levels of dynamics; a slow chromatin compartment and a fast compartment of proteins and RNA.

## Dynamic asymmetry leads to viscoelastic phase separation

**Figure 1:**
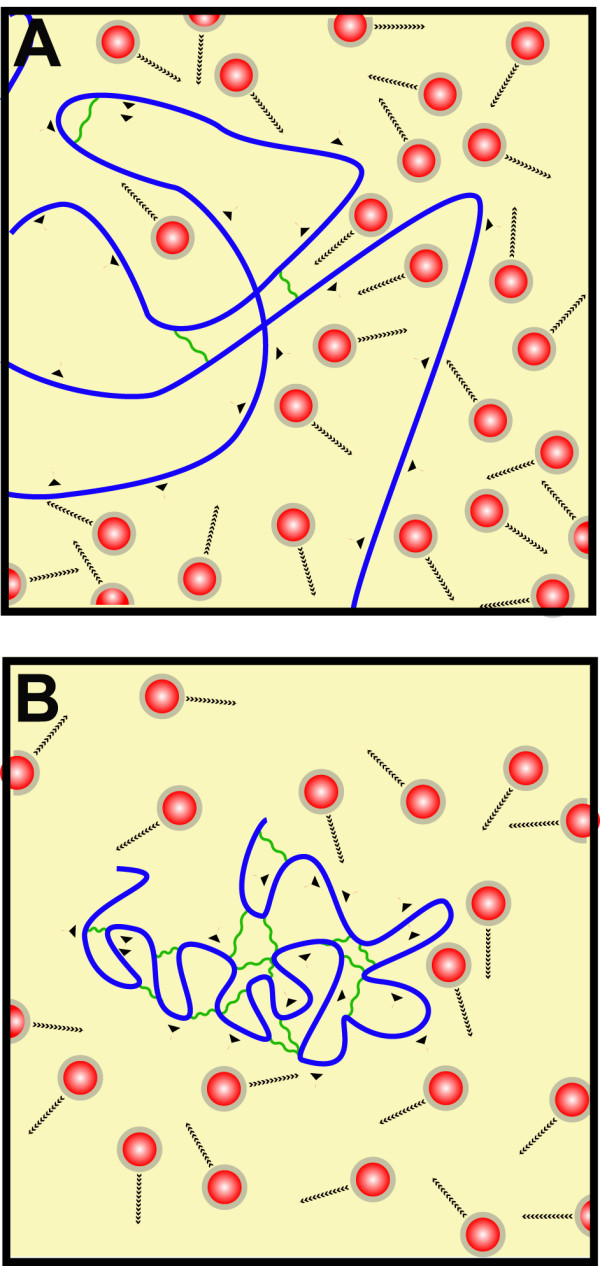
**Viscoelastic phase separation**. Viscoelastic phase separation occurs in mixtures of components with very different dynamic regimes. (A) The mixture just after mixing, with large, slow polymers (blue lines) and smaller, very dynamic molecules (red balls). Long polymers show slow movement (single arrowhead) and tend to aggregate (wavy green line) and small molecules are very dynamic (multiple arrowheads). At this time point no phase separation can be observed. (B) This shows a later snapshot of A, with more self-aggregation in the polymer, where the polymer collapse in a separated phase.

**Figure 2:**
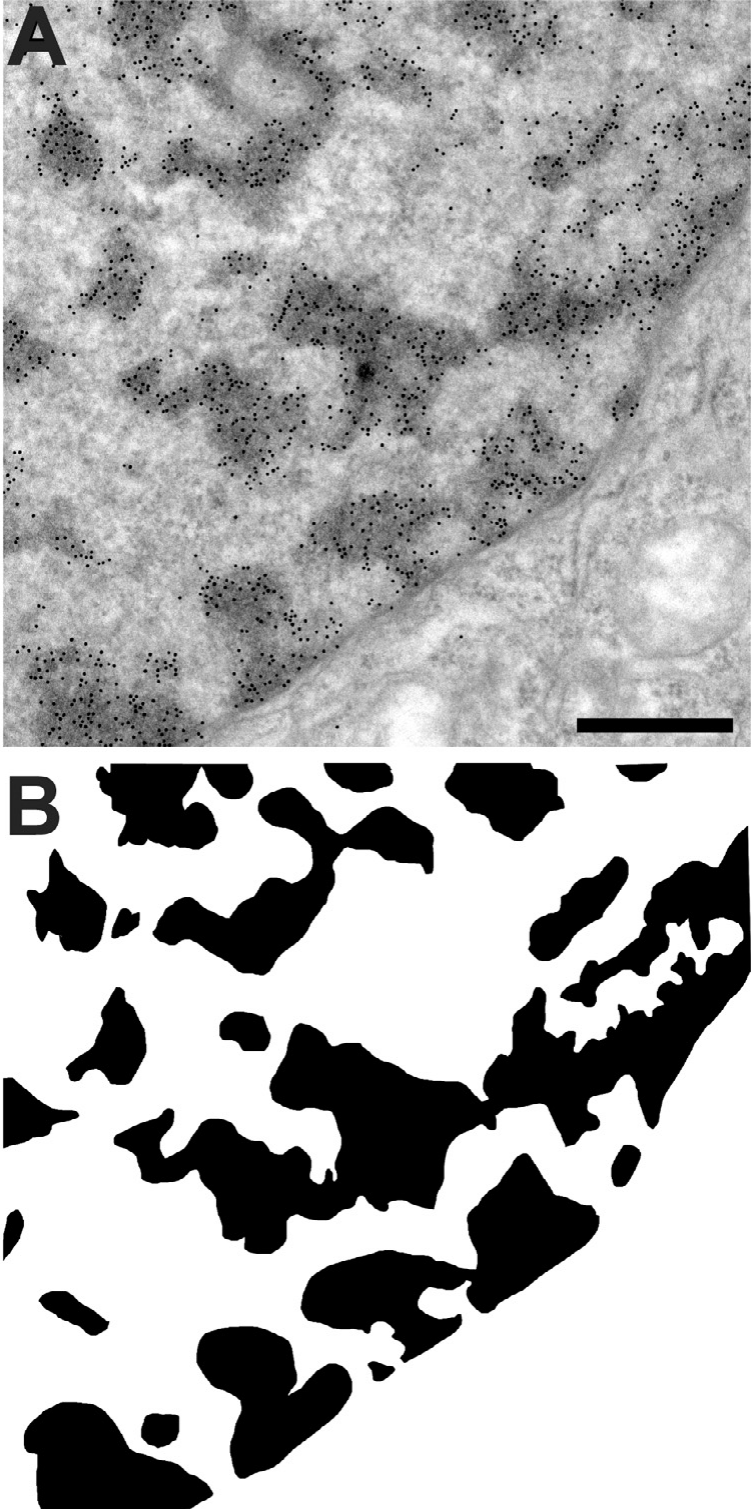
**DNA Distribution in the cell nucleus**. Distribution of DNA in HeLa cells; DNA is visualised after incorporation of 10 μM BrdU for 24 h. Cells were then processed for electron microscopy and the Br-DNA visualized after denaturation of DNA (1 M HCl, 30 min). DNA is distributed in a sponge-like pattern; this distribution is more obvious in panel B. Panel B shows a threshold image of DNA distribution. This image is very similar to the images obtained by viscoelastic phase separation [23]. Bar 500 nm.

The nucleus contains components with two very different dynamic regimes; chromatin on one hand, which shows constrained slow diffusion, and proteins and RNA on the other hand, which show diffusion at least three orders of magnitude faster. This creates a strong dynamical asymmetry, probably partly due to the difference in size between the two types of molecules. From the discipline of polymer science we know that mixtures of polymers with very different kinetic properties undergo a phenomenon known as viscoelastic phase separation [23]. That leads to the formation of two phases. One phase is a long-lived 'interaction network' (transient gel) of the slow component. The second phase, known as the 'inelastic phase', is rich in fast components and can nucleate and grow in the transient gel. The long-lived 'interaction network' can tolerate bulk stress against its volume because polymeric molecules can withstand a large degree of deformation, thus allowing individual regions in the polymer to bear mechanical stress. In contrast, free colloidal particles (inelastic phase) cannot tolerate the same stress. The phase separation becomes more pronounced when the molecules of the slow phase tend to associate [24] (Figure 1). From our understanding of the dynamic properties of the nucleus, the concept of viscoelastic phase separation could partly explain behaviour of nuclear components. The slow fluid component (DNA) cannot catch up with the deformation rate of phase separation itself and starts to behave like a viscoelastic body. This phase separation process is characterized by the generation of a sponge-like network of the slow component i.e. the chromatin, which is the result of coexistence of 'asymmetry in mobility between the two components of a mixture' and the network-forming ability originating from attractive interactions between like species [23]. In the case of chromatin the attractive forces are mediated by histone-histone interaction and ancillary proteins interacting with chromatin [25]. The theory of viscoelastic phase separation predicts that chromatin would adopt a sponge-like structure in the cell nucleus as shown in Figure 2, with fingers of chromatin penetrating the nucleoplasm full of rapidly moving protein and RNA molecules. Similar pictures to the one shown in Figure 2 have been obtained using specific DNA staining osmium-ammine B [26].

**Figure 3:**
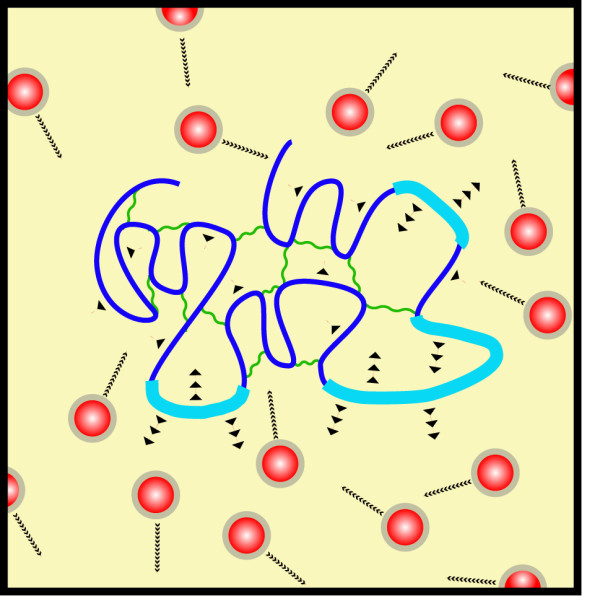
**Differences between hetero and euchromatin**. Heterochromatin (dark thin blue line) shows a high degree of self-interaction (wavy green lines) and low mobility. Decondensed euchromatin (light thick blue lines) shows a low degree of self-interaction and higher mobility than condensed chromatin.

Within chromatin itself there are different dynamic regimes that again are related to structure. Chromatin in the cell nucleus broadly appears in two forms, heterochromatin and euchromatin. The difference between these two forms resides in the transcriptional status and the morphological structure adopted. Heterochromatin is condensed and euchromatin is decondensed and is excluded from heterochromatin. How do these facts fit in with viscoelastic phase separation? Both heterochromatin and euchromatin are characterized by their specific histone methylation and acetylation patterns. Methylation of H3(K9), H3(K27) and H3(K20) are associated with the repressed chromatin state, whereas H3(K4), H3(K36) and H3(K79) methylation and/or histone acetylation have been correlated with active chromatin [27,28]. The heterochromatin proteins HP1 interact with di and tri-methylated H3(K9) in a cooperative manner due to HP1 dimerization and additional stabilizing interactions with other factors [29,30]. In this way HP1 polymerizes on heterochromatin enhancing the self-interacting properties of inactive chromatin, creating bulky chromatin masses with reduced mobility. On the other hand modifications that make the chromatin active destabilize self-interacting properties (Figure 3), which cause different mobilities of hetero and euchromatin [15]. This differential behavior of chromatin is a basic feature of viscoelastic phase separation. In this simple manner, chromatin compartmentalization can be achieved with heterochomatin aggregated in domains that exclude euchromatin, separating both phases and promoting the aggregation of active genes in particular nuclear areas as has been demonstrated [31-34].

## Macromolecular crowding

**Figure 4:**
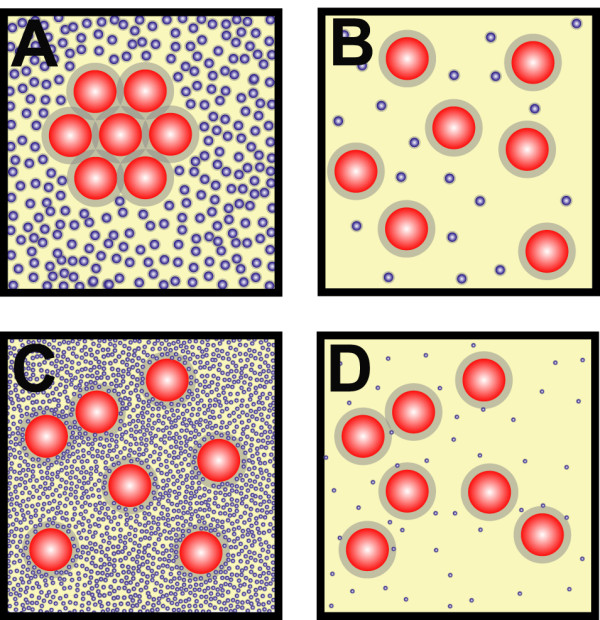
**Macromolecular crowding promotes self-association**. When molecules have the ability to self-interact, macromolecular crowding enhances their aggregation. Several situations are presented in the cartoons, two of which are common in cells (panels A and C) i.e. large macromolecules, some of which can self-associate, against a high background of non-interacting molecules. Panel A: large, self-associating macromolecules (red circles) are present alongside a high background of non-interacting molecules (blue circles) big enough to be excluded from the grey area around the big molecules, e.g. proteins or other polymers. When several red molecules self-associate the total exclusion zone decreases (grey area), increasing the area of mobility available to the blue molecules. Therefore by aggregating the red macromolecules the entropy of the system increases. Panel B: under non-crowded conditions there is no restriction on the mobility of the blue or red molecules, aggregation of red molecules results in only a small increase in entropy. Under these condition, aggregation of the red molecules is not favoured [35]. Panel C: large macromolecules present alongside a high concentration of relatively small molecules e.g. salt molecules. Panel D: similar situation to panel C, but with a low concentration of small blue molecules. In both C and D, the small molecules have free access to the entire system. Consequently there is no thermodynamic advantage for the large macromolecules to associate; therefore aggregation of large macromolecules is not affected by the concentration of the small molecules.

Around 20 to 30% of the intracellular volume is occupied by macromolecules, with concentrations reaching 200–300 mg/ml [35]. These high concentrations lead to macromolecular crowding, a process that dramatically increases intermolecular interaction rates [36,37] and can lead to segregation of macromolecules into discrete phases by demixing [38]. An illustration of the phenomenon is presented in Figure 4. Depletion interaction arises from the presence of a smaller, non-adsorbing species in a particle suspension, such as polymer molecules or other small nanoparticles. The origin of the interaction was first explained successfully by Asakura and Oosawa using the concept that the free volume available to non-adsorbing polymer molecules increases whenever two hard particles approach sufficiently close [39].

Crowding effects have been demonstrated to play an important role in formation of some protein-based nuclear compartments. It has been shown that nucleoli and PML bodies disassemble when nuclei are expanded to twice their normal volume by hypotonic treatment [2]. Reassembly of these compartments can be achieved by either returning the nuclear volume to normal in standard buffer or, alternatively, by adding inert macromolecules to expanded nuclei. In addition, the dynamic nature of compartments, with a constant exchange of macromolecules between the compartment and the nucleoplasm, is consistent with the properties of demixed phases produced by crowding [38]. The spherical/spheroid shape of nuclear bodies are typical morphologies generated by macromolecular crowding, as this globular conformation is favoured by macromolecular crowding theory [36,37]. Nevertheless, macromolecular crowding in itself is not enough to generate compartments. We know that the expression of GFP alone does not generate any kind of structure arguing for the need for some kind of self-associating properties in the constituents of the body. This has been shown to be the case in the well-studied Cajal body, where the presence of self-interacting proteins is required for the body formation and also in PML or SMN bodies [40-43]. Recently it has been shown that SUMO modification of PML components is essential for PML body formation. It seems that proteins containing SUMO-binding motifs act as a scaffold for the formation of large macromolecular complexes [44,45]. Interestingly, assigning specific functional roles to these two bodies in particular has been elusive.

It may be that these bodies are the result of pure biophysical forces and have no truly functional role as a body. In this vein, we know that chemical reactions are a function of interaction between molecules, which interact through their surfaces. Reactivity can be viewed as a problem of molecular surfaces. The surface area associated with a given mass of material subdivided into equal-size particles increases in inverse proportion to the linear dimensions of the particles. Put simply, the bigger the structure, the lower the surface area exposed. This point can be easily illustrated by example of a small molecule of 10 nm of diameter that can self-interact, building structures from 200 nm to 1 μm. Using a 10 nm monomer, 8000 and 10^6 ^molecules are respectively required to build a 200 nm or 1 μm structure. The reduction in surface of these particles in the bulk state is strikingly evident; 95 and 99% reduction, in the case of 200 nm and 1 μm respectively. The argument is obvious; when proteins accumulate in bodies their specific activity (Units of activity per molecule) become reduced by a factor proportional to the size of the body. This would support the premise that nuclear bodies are less likely to represent a functional state.

**Figure 5:**
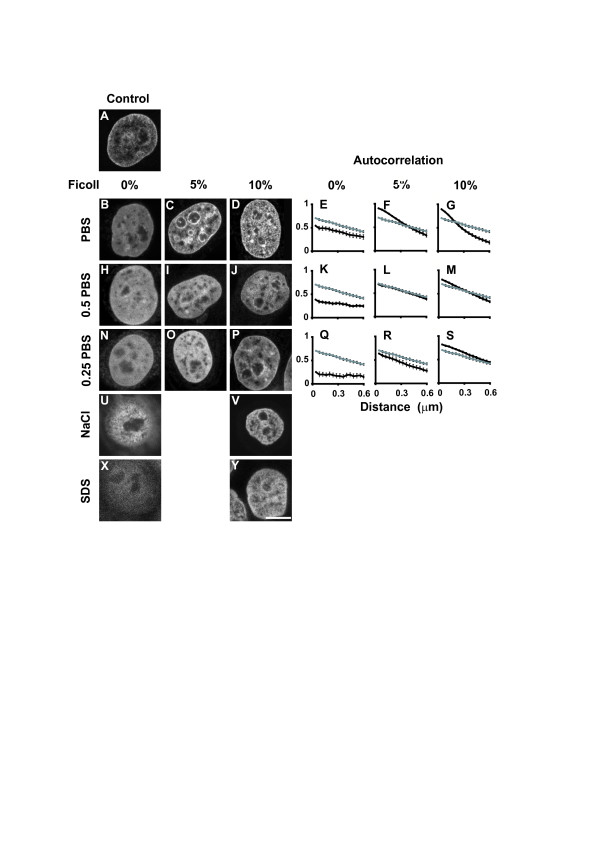
**Macromolecular crowding plays a role in chromatin condensation**. Hela cells were exposed to different concentrations of PBS and Ficoll 400. Following 10 minutes of exposure to the experimental conditions, cells were fixed with 2% paraformaldehyde, stained with 20 nM Topro 3 for 5 min, and then images were collected. Panels A to N show representative images for each condition. Autocorrelation analysis was performed as follows: intensity profiles were measured across the nucleus, taking care to avoid nucleoli. These measurements were used to compute correlation by self-reference, for a series of lag distances (unit used was 40 nm) from each point n≥ 50, and the average and standard deviation were plotted. The autocorrelation plots show the variation of correlation with the distance. Ecologists have used this type of analysis extensively, as it gives information about spatial patterns. Panel A: chromatin distribution in a non-permeabilised cell. Panels B to J: representative images illustrating cells permeabilised with 0.1% Triton and incubated for 10 minutes with buffers of different ionic strength and with different concentrations of macromolecular crowding agents. Incubation with PBS alone is sufficient to decondense chromatin (B, E), and this decondensation increases with lowering PBS concentrations (H, K and N, Q). The addition of Ficoll was able to maintain chromatin condensation and even to hyper-condense chromatin (C, D, I, J, O, P). However, to achieve conditions similar to unperturbed cells, a combination of both ionic strength and macromolecular crowding is required. Panels E, F, G, K, L, M, Q, R, S show the autocorrelation analysis of images from nuclei treated with the varying conditions represented in the picture panels. The correlogram plots show measurements from chromatin of control nuclei (grey) and nuclei exposed to the different experimental conditions (black). Values above or below control correlogram, represents hyper-condensation or decondensation respectively. This analysis is sensitive enough to detect small variations, and may be used as a quantitative test for chromatin structure. The experimental conditions that best mimic the nuclear milieu are an ionic strength of 50% PBS and 5% Ficoll 400, as shown by the overlap of both correlograms (Panels I and L). Panels U and V show the chromatin distribution in nuclei incubated with 2 M NaCl. Most of the DNA is extruded from the nuclear interior (U) but when the same experiment was performed in presence of 10 % Ficoll (V) the chromatin integrity was maintained. Panels X and Y illustrate the chromatin distribution in nuclei treated with SDS (1%), which completely destroys nuclear integrity and massively decondenses the chromatin (X). Addition of 10% Ficoll (Y) was able to preserve chromatin integrity. Bar 5 μm.

Macromolecular crowding also plays a role in chromatin structure. This is illustrated by the experiment shown in Figure 5. HeLa cells were treated with 0.1 % Triton-X-100 in order to disrupt the cell membranes thereby allowing free diffusion of molecules in and out the nucleus. Under the experimental conditions described, cells were incubated with buffers containing different concentrations of salts and/or macromolecular crowding agents. One could observe a clear relationship between macromolecular crowding agent and changes in compaction of the chromatin; the higher the concentration of macromolecular agent, the more condensed the chromatin. As expected, the ionic strength of the buffer also affected the chromatin structure, due to the strong negatively charged nature of chromatin [26]. However, alterations in chromatin structure could be reversed by the addition of macromolecular agents alone, suggesting that crowding is sufficient to return chromatin to its native integrity. This experiment illustrates the role of macromolecular crowding in maintaining chromatin compartmentalization and the interplay with ionic conditions.

## Colloidal properties: The speckle compartment as an example

A distinctive compartment, which illustrates another type of physical force operating in the nucleus, is the IGC. Within mammalian cells, pre-messenger RNA splicing machinery is found in a compartment referred to as the speckles, splicing factor compartment, SC-35 domains or IGC. By fluorescence microscopy these nuclear speckles are seen as irregular shaped bodies located at interchromatin regions. When nuclear speckles are examined by electron microscopy, they can be seen to be composed of clusters of interchromatin granules, measuring 20–25 nm in diameter [46]. They contain numerous factors involved in RNA synthesis and processing and seem to be involved in assembly or modification of these factors [46,47]. Unlike other nuclear bodies, the nuclear speckle compartment is known to be positionally stable. Time-lapse observations of nuclear speckles in living cells have shown that their position is maintained over many hours [48,49].

**Figure 6:**
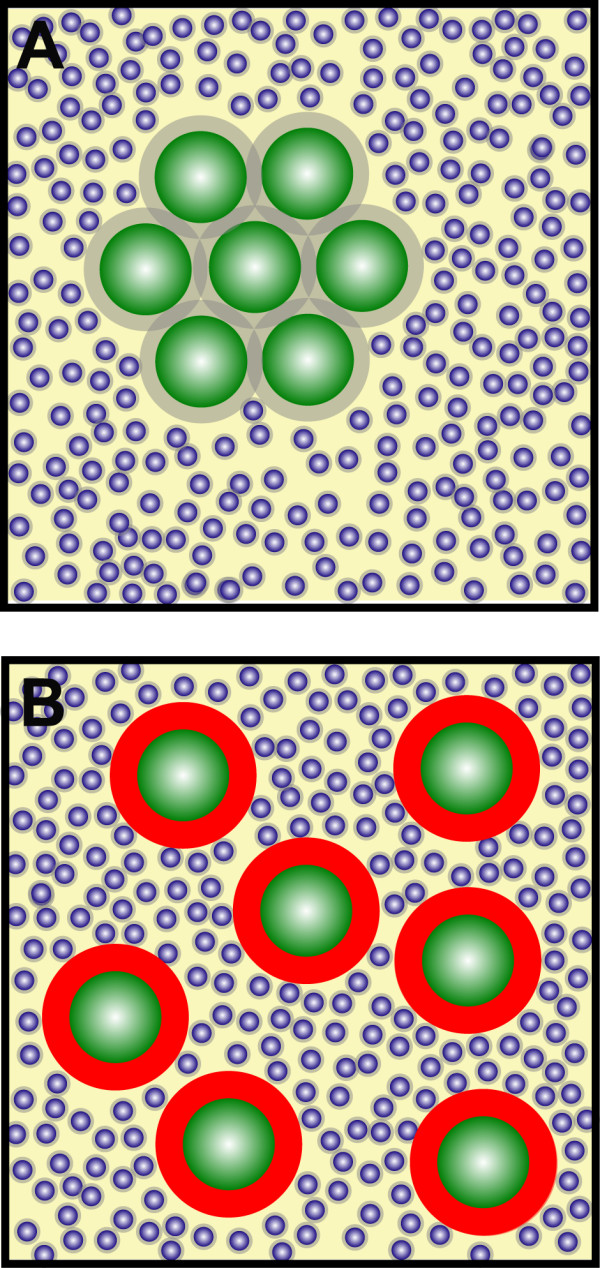
**Colloidal particles**. SC-35 speckles are formed by granules that behave as colloidal particles. (A) When the particles are not electrically charged (green circles with grey halo), excluding volume effects are the dominant force determining structure. (B) When particles become charged (phosphorylated, red halo around green circles), repulsive forces are stronger than excluding volume forces and the interchromatin granule clusters become destabilized.

The speckle compartment could be viewed as a long-lived interaction network like chromatin, because their dynamic properties are different from the freely diffusible molecules of the nucleoplasm. From a biophysical standpoint, speckles could also be viewed as a 'colloidal suspension of IGC, colloids being defined as particles in the range of 1 nm to 1 μm [50]. One of the physical properties of colloidal suspensions is that upon addition of non-absorbing polymers, phase separation can be induced (Figure 6). This is due to polymer-induced volume depletion, which increases attraction between colloidal particles [51]. When colloids are physically close, the region around the colloid from which polymers are sterically excluded can overlap. The resulting unbalanced osmotic force causes attractive interactions between colloidal particles [39]. This phenomenon occurs on neutrally charged particles, as electrostatic charge can be sufficient to destabilize clusters formed by polymer-induced volume depletion. Consistent with these facts, we know that exchange of granules on the IGC occurs at the periphery of the IGC and is regulated by phosphorylation, probably by altering the self-interacting properties of these granules [52]. Hyper-expression of the kinases that phosphorylate these granules, hence changing their charge, destroys the IGC [53], which fits with the colloidal model. In addition, treatment of cells with kinase inhibitors results in bigger IGC and in inhibition of the dynamic movements on the periphery of IGC [48].

IGC is a reversible flocculate of colloidal particles and as such structure is irregular and bulky, which makes it practically immobile, in perfect agreement with the documented possitional stability of IGCs [48].

The predominant force determining structure in the case of nuclear speckles seems to be the colloidal properties of interchromatin granules.

## The nuclear matrix: another manifestation of colloidal properties of nuclear constituents

A debatable compartment is the nuclear matrix. This compartment is visible after extensive extraction using detergents, high salt solutions and treatment with nucleases. The nuclear matrix is a fibro-granular network with a complex protein composition [54,55]. Many scientists have tried to visualize this structure in un-extracted cells by light or electron microscopy, but these attempts have been unsuccessful. They have tried to express these proteins tagged with GFP or using specific antibodies to visualize the nuclear matrix, but all these efforts have failed to demonstrate the existence of nuclear matrix. For these reasons many scientists believe that the nuclear matrix is no more that an experimental artifact that has nothing to do with the physiology of the cell. Moreover, many of the proteins implicated in the nuclear matrix formation are known to be highly dynamic which would appear to be in direct opposition of the idea of a nuclear skeleton.

I believe that some important clues to the solution of this controversy lie in the physical properties of macromolecules. Almost every single macromolecule found in the cell nucleus is a colloid (colloids have sizes ranging from 1 nm to 1 μm). Colloidal particles have a very distinctive set of properties. Their solubility properties strongly depend on the ionic conditions of the medium; just changing the ionic conditions means that the solubility of colloids changes. Colloidal particles in solution carry a electric charge, which have dual origin: the pH and ionic composition of the medium [50]. The pH will determine the ionization of the radical groups of the amino-acid chains (in the case of proteins) or the phosphate groups (in the case of RNA). The ions present in the medium will be adsorbed on to the surface of the colloidal particle and together with the pH will determine the solubility properties of the particle under physiological conditions. We know the range of physiological pH inside the nucleus, but we are far from an exhaustive and detailed knowledge of the ionic composition. The coagulation of colloidal particles is very well known for more than a century – Faraday in 1856 described the coagulation of colloidal particles by addition of ions to the media- [50]. Therefore one could imagine that nuclear matrices are in fact aggregations of colloidal particles, which precipitate giving the beautiful structures seen in nuclear matrix preparations. In this way when ribonucleoproteins hnRNP A2 and hnRNP B1, at low protein concentration, are exposed to high ionic strength buffers, they associate in regular helical filaments ranging in length from 100 nm to 10 μm with diameters from 7 to 18 nm. However, when the protein concentration was raised, the filaments rapidly aggregated forming thicker filamentous networks that look like the fibrogranular structures of nuclear matrices [56]. For these reasons it is not surprising to find MARs (Matrix Attachment Regions) in nuclear matrix preparations, because these MARs are transcriptionally active, therefore the RNA bound to RNPs emerging from them will aggregate in the artefactual nuclear matrix preparation, dragging the RNA pol II associated with the DNA (MARs).

## Functional implications of viscoelastic phase separation and macromolecular crowding

Understanding the mechanisms of compartmentalization is essential to understanding nuclear processes and their control. By separating the non-specific biophysical effects of phase separation and macromolecular crowding from the truly specific interactions, one can really begin to appreciate nuclear organization and its relation to function. Spherical nuclear bodies can be explained by macromolecular crowding effects, but not the sponge-like structure adopted by chromatin, which is explained by viscoelastic phase separation. If one accepts that nuclear components are subject to viscoelastic phase separation, the implications of such a phenomenon can be addressed.

If phase separation does occur in the living cell then interactions between the phases, namely the chromatin and protein compartments, should be limited to the interface between the two. This is indeed what we observe. It is well known that colloidal particles (eg. proteins, spliceosomes, RNPs) partition at the interface in systems where phase separation occurs [57,58] – this will be enhanced in proteins with an affinity for DNA. Proteins adsorbed at the interface would exhibit reduced Brownian movement. This could be an alternative interpretation to models that propose chromatin associated proteins first associate with nonspecific sequences of DNA at low affinity and then diffuse along the DNA in one dimension searching for a "bona fide" binding site [59]. Nonspecific binding of these proteins to the DNA could account for the population demonstrating intermediate dynamics between freely diffusible and fully bound [60].

**Figure 7:**
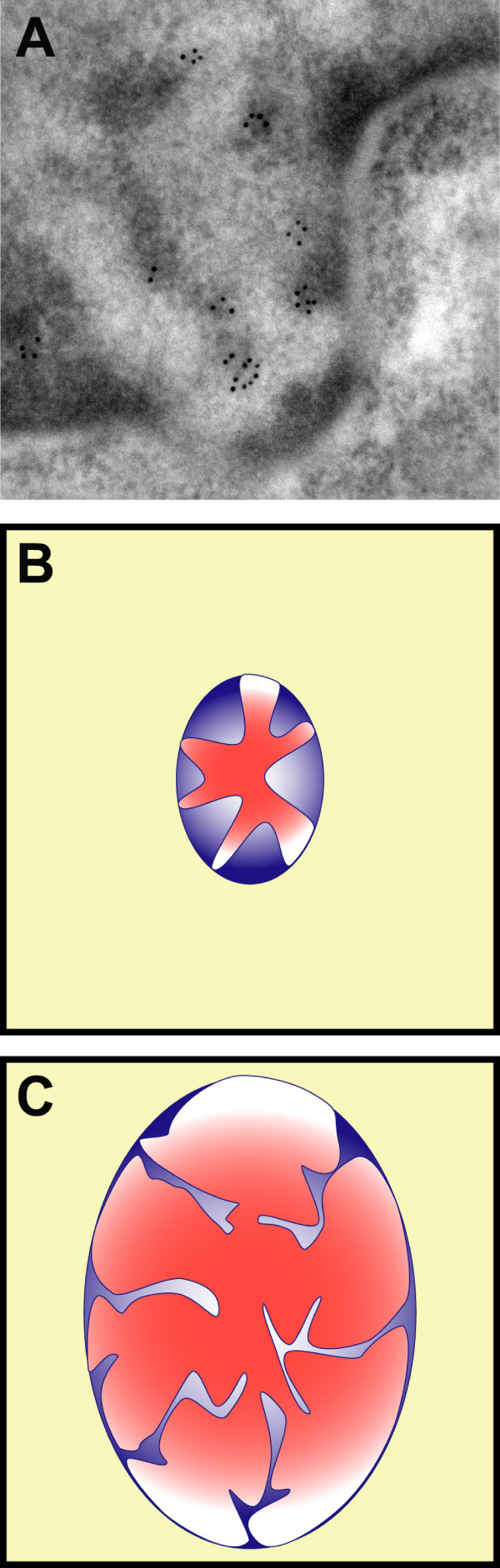
**Chromatin interphase**. Transcription occurs at the interface of chromatin. (A) Electron micrograph showing transcription by RNA pol II (gold particles) at the border of condensed chromatin masses. Figure reproduced from reference 52, with permission from Springer. Activation of transcription proceeds with nuclear volume change. The amount of chromatin (blue) is stable and only the dynamic phase (red) can be changed. As a result by increasing the amount of proteins/RNA, the nuclear volume changes and also the interface between DNA and proteins/RNA, where transcription occurs. (B) Cell with low level of activity (small interface). (C) Very active cell (Large interface).

Another example of this phenomenon is transcription, where in order to transcribe the information encoded on DNA, RNA polymerases need to interact with the DNA. Transcription in the cell nucleus occurs at the interface between condensed masses of chromatin and the interchromatin space [61,62], (Figure 7). Viscoelastic phase separation also predicts that the interface between the fast and slow component, must change in a manner proportional to the alteration in either fraction. For example, if the fast phase increases, the slow phase (DNA) will become more extended, increasing the interface surface. Likewise, a decrease of fast phase will be associated with a compaction of the slow moving phase and a reduction in the interface surface [23]. Again this is precisely what happens in the nucleus. Activation and repression of transcriptional activity are associated with changes in the kinetically fast phase, i.e interchromatin space (ICS). Transcription activation has always been linked with an increase in the ICS. Indeed, the first manifestation of reactivation was a volume enlargement of the erythrocyte nucleus followed by an increase in transcription [63]. When HeLa cells were fused with quiescent chicken erythrocytes, the result was reactivation of the chicken nuclei within minutes after the increase of the nuclear volume [63]. Lymphocyte activation with Concavalin A proceeds with up to six fold increase in nuclear volume, due to a near 10-fold increase in the ICS region. This increase in ICS is paralleled with transcriptional activation [64]. On the other hand, transcriptional shut down is accompanied by extrusion to the cytoplasm of ribonucleoproteins, splicing factors, and other nuclear proteins into structures called HERDS that eventually become degraded, with nuclei shrinking dramatically during the process [65]. Therefore, activation of transcription involves an increase in the interface where transcription is occurring [61]; and transcription repression a decrease of the interface.

In conclusion, I propose the hypothesis that nuclear compartmentalization is the result of the physical forces operating in the cell nucleus. In this way the different structures observed will depend on the characteristics of the compartment components. When viscoelastic phase separation is the prominent force, the compartment generated will have sponge like structure. When no strong dynamic asymmetry applies to the components of the compartment and self-association occurs, then macromolecular crowding is the driving force generating spherical structures. If no dynamic asymmetry applies to the components of the compartment and they do not self-associate, then no phase separation will operate and the compartment will look unstructured. If colloidal properties are predominant, the structure will behave like a colloidal gel.

The concepts of viscoelastic separation and macromolecular crowding appear to be consistent with the literature in the field of nuclear structure and function. Appreciation that biophysics has an important role to play in nuclear organisation will doubtless lead to a better understanding of the functions of the nucleus and more importantly give insight what may be happening when these processes go awry.
